# An examination of trends in antibiotic prescribing in primary care and the association with area-level deprivation in England

**DOI:** 10.1186/s12889-020-09227-x

**Published:** 2020-08-03

**Authors:** Katie Thomson, Rachel Berry, Tomos Robinson, Heather Brown, Clare Bambra, Adam Todd

**Affiliations:** 1 Population Heath Sciences Institute, Faculty of Medical Sciences, Newcastle University, Newcastle upon Tyne, UK; 2 Fuse – the UKCRC Centre for Translational Research in Public Health, Newcastle upon Tyne, UK; 3 NHS County Durham Clinical Commissioning Group, Durham, UK; 4 School of Pharmacy, Faculty of Medical Sciences, Newcastle University, Newcastle upon Tyne, NE2 4AX UK

**Keywords:** Antibiotics, Prescribing, Deprivation, General practice, Health inequality

## Abstract

**Background:**

Internationally, there are growing concerns about antimicrobial resistance. This has resulted in increased scrutiny of antibiotic prescribing trends – particularly in primary care where the majority of prescribing occurs. In England, antibiotic prescribing targets are set nationally but little is known about the local context of antibiotic prescribing. This study aimed to examine trends in antibiotic prescribing (including broad-spectrum), and the association with area-level deprivation and region in England.

**Methods:**

Antibiotic prescribing data by GP surgery in England were obtained from NHS Business Service Authority for the years 2014–2018. These data were matched with the Index of Multiple Deprivation (IMD) 2015 at the Lower Layer Super Output Area level Lower Layer Super Output Area (LSOA) level. Linear regression methods were employed to explore the relationship between antibiotic use and area-level deprivation as well as region, after controlling for a range of other confounding variables, including health need, rurality, and ethnicity.

**Results:**

Over time, the amount of antibiotic prescribing significantly reduced from 1.11 items per STAR-PU to 0.96 items per STAR-PU – a reduction of 13.6%. The adjusted models found that, at LSOA level, the most deprived areas of England had the highest levels of antibiotic prescribing (0.03 items per STAR-PU higher). However, broad spectrum antibiotic prescribing exceeding 10% of all antibiotic prescribing within a GP practice was higher in more affluent areas. There were also significant regional differences – with the North East and the East of England having the highest levels of antibiotic prescribing (by 0.16 items per STAR-PU).

**Conclusion:**

Although antibiotic prescribing has reduced over time, there remains significant variation in by area-level deprivation and region in England – with higher antibiotic prescribing in more deprived areas. Future prescribing targets should account for local factors to ensure the most deprived communities are not inappropriately penalised.

## Background

Antimicrobial resistance poses a significant challenge to modern day medicine and has been described by the World Health Organisation (WHO) as a global health security threat [1]. Since the 1940s, over 140 antibiotics have been developed for humans where they have had huge benefits in treating infectious disease. However, this ‘golden age’ of antibiotics appears to be over – with only two novel classes of antibiotics launched in the last 30 years [2]. As bacterial resistance becomes more frequent, there has been a strategic focus toward developing antimicrobial stewardship polices in order to minimise the burden of antimicrobial resistance [3]. In response to these challenges, the WHO has set up a taskforce on antimicrobial resistance with the aim of developing national and regional action programs [1]. In response, the English Department of Health has developed an antimicrobial resistance strategy [4], which has the overall aim of reducing the use of antibiotics when it is safe and appropriate to do so: the target is to reduce the levels of inappropriate antibiotic prescribing by 50% by 2020 [5].

In addition to targets for overall prescribing, particular focus has also been placed on reducing the prescribing of specific broad spectrum antibiotics, such as co-amoxiclav, the cephalosporins, and the quinolones, owing to their potential to cause severe adverse effects, such as *Clostridium difficile* infection. Optimizing prescribing practices is considered a key component of this strategy, which highlights the importance of understanding antibiotic prescribing patterns across different areas, although very few studies have reported this; the majority of research has focused on antibiotic prescribing in subsets of practices [6, 7] or across a single region [8]. Previous work by Curtis and colleagues has shown that, in England, higher prescribing rates of antibiotics are associated with more deprived areas, as well as greater GP practice size, a higher proportion of older or younger patients, and ruralness [9]. It is not known, however, if these higher prescribing rates are driven by health need (e.g. people living in deprived areas are more likely to have health conditions that are associated with the use of antibiotics) or other factors, such as health seeking behavior or quality of GP services given that the majority of antibiotics are prescribed in primary care [10].

There is a need to understand the *local* nature of antibiotic prescribing by primary care in England – especially in relation to area-level deprivation. It is well established that health need varies by area-level deprivation with higher rates of morbidity and mortality in the most deprived neighbourhoods [11]. For example, in England there are life expectancy gaps of up to 9 years for men and 7 years for women between the most and least deprived areas [11]. It is likely therefore that antibiotic prescribing will reflect this health need and be higher in the more deprived neighbourhoods. Currently, English antibiotic targets do not recognise deprivation as a driver of prescribing, as the targets only tend to control for age and sex. It is crucial therefore that we explore the relationship between area-level deprivation and antibiotic prescribing in order to inform future prescribing targets in England. It is important that the targets do not adversely penalise areas with higher health need and thus potentially widen health inequalities.

This study, therefore, aimed to: (1) examine the association between antibiotic prescribing and area-level deprivation; (2) analyse the proportion of broad spectrum antibiotics (including co-amoxiclav, the cephalosporins and the quinolones) prescribed by area-level deprivation; and whether (3) geographic region influences prescribing rates.

## Methods

### Prescribing data

Antibiotic prescribing data were obtained from the NHS Business Services Authority ePACT2 system (NHSBSA Copyright 2018). The data were downloaded for English General Practices (GPs) for the 4 years from April 2014–March 2015 to April 2017–March 2018. All GP practices open for the entire year were included in the analysis. Antibiotic prescribing was measured according to the items per STAR-PU (Specific Therapeutic Group Age-sex weightings Related Prescribing Units) weighting, which shows the amount of prescription items that have been prescribed, compared to what would be anticipated given the number and characteristics of patients registered in the practice. The numerator is the total number of prescription items for antibacterial drugs (as defined by the British National Formulary, Chapter 5.1), and the denominator is the total number of oral antibacterial drugs (as defined by British National Formulary, Chapter 5.1) ITEM based STAR-PU. As such, it is possible to use STAR-PUs, instead of the number of patients, to allow for comparisons between General Practices. STAR-PUs adjust for age and sex in prescribing, but do not control for health need or area-level deprivation in the weighting. Lower values of items per STAR-PUs indicate less oral antibacterial prescribing. In addition, we also report when broad spectrum antibiotic prescribing (namely co-amoxiclav, cephalosporins and quinolones) exceeds 10% of all antibiotics prescribed within a GP practice; the threshold of 10% is based upon NHS England targets [12]. This measure looks at the quantity of these drugs (as a percentage), versus the total number of antibiotics prescribed. Practice codes were cross-referenced with the NHS Digital GP Practice Database [13], and any practices that were not open for the full relevant year were excluded. Practices not classified as GP practices (e.g. out of-hours or specialist prescribing) were also excluded from the analysis. To account for outliers in the prescribing data owing to potential incorrect coding of GP practices in the database, we excluded the top and bottom 1% of items prescribed by items per STAR-PU and the percentage of broad spectrum antibiotics.

Overall, 29,631 GP surgeries were included in the analysis for antibiotic prescribing over the 4 years for which we have data (representing 7700 unique surgeries). Seven GP surgeries were excluded as they did not have a corresponding Index of Multiple Deprivation (IMD) decile, and a further 606 were removed as they represented the top and bottom 1% of antibiotic items per STAR-PU data (see histogram in Supplementary Figure 1 to illustrate before and after comparisons). There is an approximate negative linear correlation between the concentration of GP practices in England and IMD decile. For the most recent data (2017–18), 1086 GP practices were located in IMD decile 1 compared to just 437 in IMD decile 10 (see Supplementary Figure 2 for graphical representation).

### Deprivation and regional data

The location of the GP practice based on their address from the reference database was matched to the corresponding Lower Layer Super Output Area (LSOA). Deprivation data was derived from the IMD 2015 produced by the Department for Communities and Local Government. This index, constructed from seven domain indices were combined to produce an overall measure of deprivation, which ranks every LSOA in England from 1 (most deprived) to 32,844 (least deprived area). Ranks were converted to deciles to visually illustrate antibiotic prescribing by GPs in different levels of deprivation whereby 1 represents the most deprived areas and 10 represents the least deprived. The IMD 2015 data is used for all years in subsequent analysis as it is produced once every 4/5 years. Using their LSOA locations, the GP practices were also put into one of the nine English government office regions: North East, North West, Yorkshire & the Humber, London, East of England, West Midlands, East Midlands, South East and South West.

### Confounding variables

A number of variables were also included in our model specifications. We included ethnic composition, as it has previously been shown that different ethnic groups have different antibiotic consumption patterns [14], these data were obtained from the Office of National Statistics (based on the 2011 census data available from: https://www.nomisweb.co.uk) to determine the ethnic composition of each LSOA. We also included urbanity in the model (produced from the 2011 census), as it has previously been shown that GPs working in rural areas were less likely to delay prescribing of antibiotics [9]. In keeping with previous studies [15, 16], the measure of urbanity, based on the Department for Environment and Rural Affairs’ rural/urban classification, uses a twofold grouping: (1) urban; (2) rural. To account for health need, we included the prevalence of COPD and diabetes at GP practice level downloaded from the Quality and Outcome Framework [17]. We used these conditions as a proxy indicator of health need, as they are associated with increased incidence of bacterial infections requiring the use of antibiotics [18, 19].

### Statistical analysis

To assess the impact of deprivation on the level of antibiotic prescribing, we used multiple bar graphs reporting items per STAR-PU and the proportion of broad spectrum antibiotics in each deprivation decile by year. To complement this graphical analysis, we also used a random-effects linear regression model to estimate the association between deprivation and antibiotic prescribing. This was seen as the most appropriate model specification, as the antibiotics prescribed per STAR-PU were approximately normally distributed (after removing the top and bottom 1% of data). The parameters were calculated using the XTREG command in the statistical software package Stata [20]. The parameter estimates in all models are given with 95% confidence intervals, with standard errors clustered at the individual level. A visual inspection of a plot of the model residuals indicated that homoscedasticity was unlikely to influence the results, while the variance of inflation (VIF) ratio was low (2.3), implying that multicollinearity was also unlikely to be problematic. There was relatively little missing data (~ 5%), with the results from the test proposed by Verbeek and Nijman [21] indicating that small amount of non-response across the four waves of data was unlikely to be non-random.

## Results

### Antibiotic prescribing by area level deprivation

**Fig. 1:**
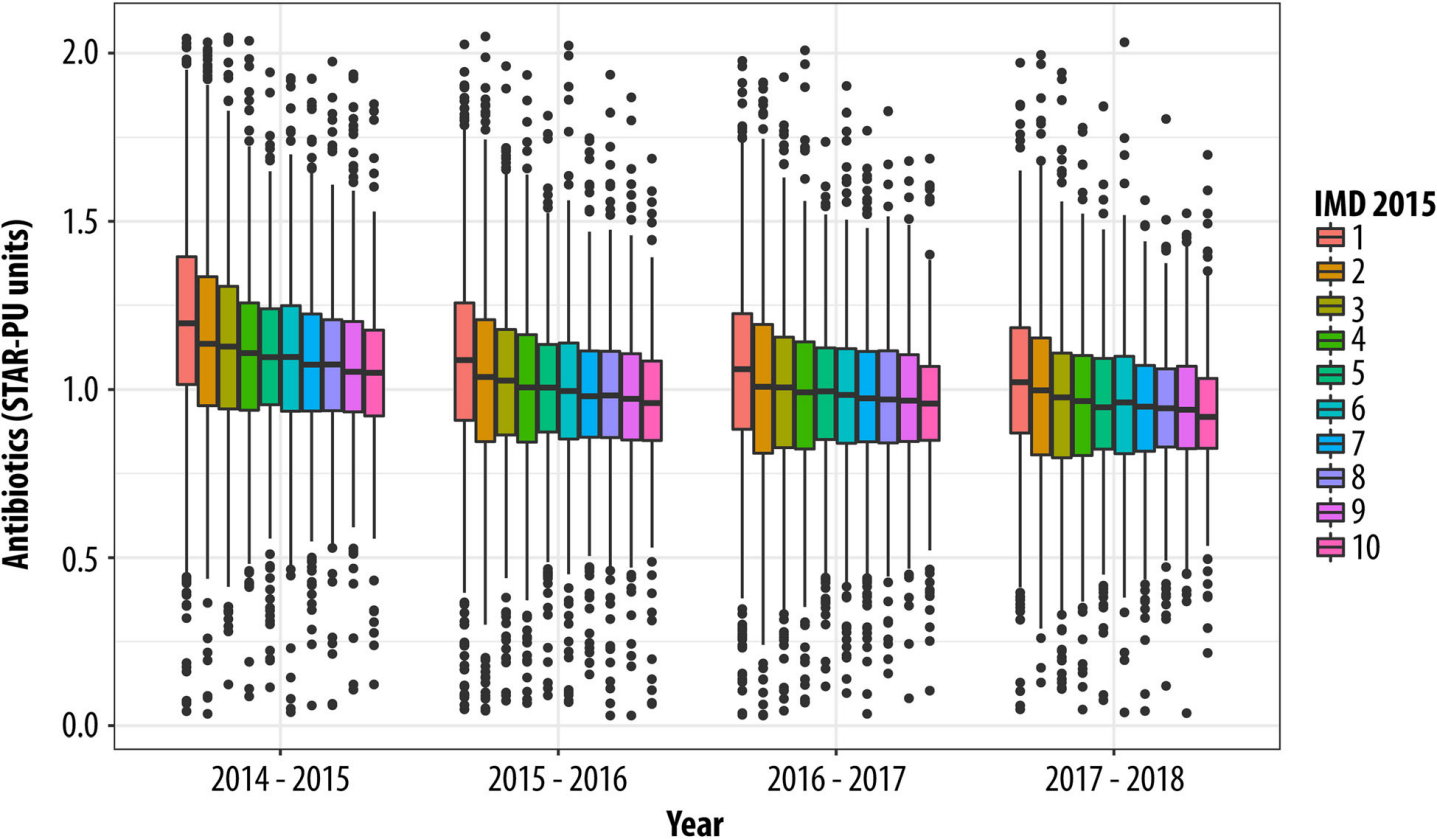
GP practice antibiotic prescribing according to items per STAR-PU (mean) by IMD decile in England (1 most deprived, 10 least deprived)

Overall, the prescribing of antibiotic items decreased from 1.11 items per STAR-PU in 2014 to 0.96 items per STAR-PU in 2018 – a reduction of 13.5%. The reduced antibiotic prescribing was more pronounced in areas of high deprivation: a reduction of 0.17 items per STAR-PU (a reduction of 17.0%) was observed for GP surgeries located in the most deprived areas (IMD-1), compared with a reduction of 0.12 items per STAR-PU (a reduction of 13.1%) for GP surgeries located in the least deprived areas (IMD-10). Antibiotic prescribing in England over time by area-level deprivation is illustrated by Fig. 1.

### Prescribing of co-amoxiclav, cephalosporins, and quinolones by area level deprivation

**Fig. 2:**
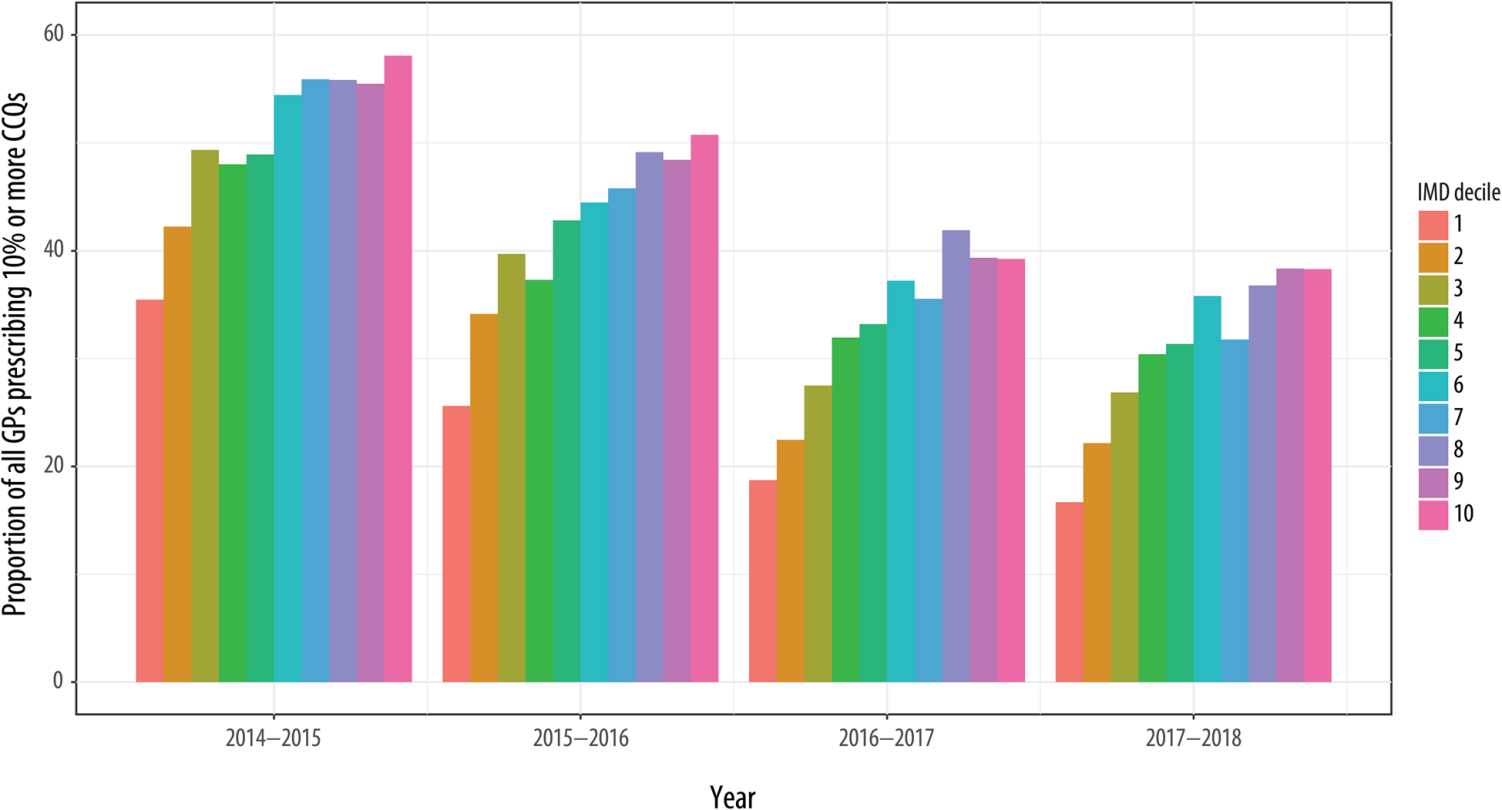
The proportion of GP surgeries that prescribe more than 10% broad spectrum antibiotics (CCQs: co-amoxiclav, cephalosporins, and quinolones) by IMD decile (1 most deprived, 10 least deprived)

Overall, the proportion of GP surgeries which prescribed over the target of 10% broad-spectrum antibiotics (co-amoxiclav, cephalosporins, and quinolones) in terms of total antibiotics prescribed has decreased from 48.4% in 2014 to 29.0% in 2018. In contrast to overall antibiotic prescribing, GP surgeries located in the most affluent areas generally had a higher proportion of prescriptions for broad spectrum antibiotics when compared with GP practices located in the most deprived areas (Fig. 2). This finding was evident for all years for which we had data, although the proportion of GP surgeries prescribing over the 10% target did decrease each year. For example, in 2014, the prescribing was 35.5% (for the most deprived areas, IMD-1) and 58.1% (for most affluent areas, IMD-10), while in 2018, the prescribing was 16.7 and 38.3% for the most deprived and affluent areas, respectively.

### Confounding factors associated with antibiotic prescribing

Two models were developed to understand the factors which drive antibiotic prescribing from English GP surgeries (Table 1). Model 1 demonstrated that living in the most deprived decile (compared to the least deprived) was associated with an increased likelihood of antibiotic prescribing by 0.15 items per STAR-PU, and a clear gradient was observable across most of the deprivation deciles. Deprivation was a significant predictor of increased antibiotic prescribing in all IMD 2015 deciles (deciles 1–8, *p* < 0.01; decile 9, *p* < 0.05).

**Table 1:** Association between antibiotic prescribing and area-level deprivation in England. Robust standard errors in parentheses **p < 0.01, *p < 0.05

MODEL NUMBER	(1)	(2)
VARIABLES	Excludes measures of ‘need’	Includes measures of ‘need’
	Coefficient (Robust Standard Error)	Coefficient (Robust Standard Error)
**LSOAs IMD**		
1 (Most Deprived)	0.15 (0.01) **	0.03 (0.01) *
2	0.13 (0.01) **	0.03 (0.01) *
3	0.11 (0.01) **	0.03 (0.01) *
4	0.08 (0.01) **	< 0.00 (0.01)
5	0.06 (0.01) **	< 0.00 (0.01)
6	0.06 (0.01) **	0.01 (0.01)
7	0.04 (0.01) **	< 0.00 (0.01)
8	0.03 (0.01) **	< 0.00 (0.01)
9	0.02 (0.01) *	< 0.00 (0.01)
10 (Least Deprived)	Reference	
**Ethnicity**		
% White	< 0.01 (< 0.01) **	< 0.01 (< 0.01) **
**Rurality**		
Rural	Reference	
Urban	−0.04 (0.01) **	−0.02 (0.01) **
**Region**		
London	Reference	
East of England	0.20 (0.01) **	0.16 (0.01) **
East Midlands	0.14 (0.01) **	0.09 (0.01) **
North East	0.25 (0.01) **	0.16 (0.01) **
North West	0.21 (0.01) **	0.14 (0.01) **
South East	0.13 (0.01) **	0.11 (0.02) **
South West	0.09 (0.01) **	0.05 (0.01) **
West Midlands	0.15 (0.01) **	0.09 (0.01) **
Yorkshire and Humber	0.18 (0.01) **	0.11 (0.01) **
**Time**		
2014–2015	Reference	
2015–2016	−0.10 (< 0.01) **	−0.11 (< 0.01) **
2016–2017	− 0.12 (< 0.01) **	−0.13 (< 0.01) **
2017–2018	−0.16 (< 0.01) **	−0.18 (< 0.01) **
**Health need**		
Diabetes prevalence (%)		0.03 (< 0.01) **
COPD prevalence (%)		0.05 (0.01) **
**Wald Chi**^**2**^**Statistic**	9210.16	9880.89
**R**^**2**^	0.22	0.32
**Observations**	28,809	28,809

When additionally adjusting for a proxy indicator of health need in Model 2, it was shown that living in the most deprived decile (compared to the least deprived) was still associated with increased antibiotic prescribing – albeit to a lesser extent, but it was only significant in the three most deprived deciles (*p* < 0.05). The model also showed that, over the 4 years for which we have data, all antibiotic prescribing has reduced by 0.18 items per STAR-PU (*p* < 0.01). GP surgeries located in areas where there were greater proportion of people of white ethnic origin (p < 0.01), and those found in rural areas (p < 0.01) also showed higher rates of antibiotic prescribing. Finally, compared to London, GP surgeries located in all other areas of England had significantly higher rates of antibiotic prescribing – with the East of England and North East of England having the highest levels (by 0.16 items per STAR-PU).

## Discussion

This paper adds to the growing evidence base exploring antibiotic prescribing trends in England. It is the first though to consider antibiotic prescribing using a proxy indicator of health need, and it is also the first to explore the prescribing of broad-spectrum antibiotics (co-amoxiclav, cephalosporins, and quinolones) as a percentage of total antibiotic prescribing by area-level deprivation. We identified three key findings that will be of importance to healthcare policy discussions around antimicrobial resistance and prescribing targets: (1) there were significant inequalities in antibiotic prescribing by deprivation – with GP surgeries located in the most deprived areas having the highest levels of antibiotic prescribing (even when adjusting for a proxy indicator of health need); (2) there is also variation in the prescribing of broad spectrum antibiotics (co-amoxiclav, cephalosporins, and quinolines) with higher proportions of broad spectrum antibiotic prescribing occurring in more affluent areas; (3) there was also significant regional variation in antibiotic prescribing – with the highest levels of antibiotic prescribing observed in the East and North East of England.

Area deprivation has multiple influences on health so the pathways linking deprivation and inequalities in antibiotic prescribing patterns are complex. There is a large international literature on the relationship between health and place which suggests that geographical health inequalities exist as a result of both the characteristics of places (in terms of infrastructure and services, social factors and the physical environment) and of the people who live there (e.g. ethnicity individual level socio-economic status) [11]. In terms of antibiotic prescribing, the most likely pathways linking deprivation to health system characteristics include healthcare access and quality, as well as the health needs of the population. So, the higher rates of antibiotic prescribing in the most deprived areas may reflect differences in GP prescribing behaviours – more deprived areas in England have lower GP provision per head of population than the least deprived and so are more reliant on locum doctors who may be more inclined to prescribe antibiotics [22, 23]. Additionally, there may be differences between symptom severity in patients living in different areas. Our proxy indicator of health need accounted for two key conditions (diabetes and COPD) but not the severity of these illnesses. Further, our adjustment of health need did not take into account other illnesses or conditions for which antibiotics might be prescribed (such as immunosuppression, or recurrent urinary tract infections). In terms of broad spectrum antibiotic prescribing being higher in more affluent areas, the reasons for this are not clear - although it has been previously shown that people accessing out-of-hours primary care services typically receive more broad-spectrum antibiotics compared to when they access in-hours primary care services [24]. Living in an affluent area might, therefore, influence how primary care services are accessed, and, ultimately, the type of antibiotic prescribed; for example, people living in affluent areas may be able to navigate the healthcare system easier [25] and access emergency GP appointments, compared to people living in deprived areas. This should be explored in future studies.

Our broad findings are in keeping with previous research. As mentioned previously, the study by Curtis and colleagues, who described antibiotic prescribing trends across England for the years 1998 to 2017, showed that there was significant geographical variation in prescribing: at a Clinical Commissioning Group (CCG) level, the variation in overall antibiotic prescribing was two-fold, while for cephalosporin prescribing, the variation was seven-fold [9]. The work also showed that higher prescribing trends were associated with a greater GP practice size, the proportion of patients greater than 65 years, or less than 18 years, ruralness and deprivation [9]. Similarly, Covvey and colleagues evaluated antibiotic prescribing trends in Scotland, and concluded that higher rates of antibiotic prescribing were found in areas of higher deprivation, as measured according to the Scottish Index of Multiple Deprivation (SIMD) [26]. This study also assessed antibiotic prescribing by antibiotic class stratified by SIMD quintile: the authors showed that the prescribing of quinolones, cephalosporins and other beta-lactams, was generally higher in more deprived areas. Although this study did not look at the proportions of broad-spectrum antibiotic prescribing as we did, this result seems to be in contrast to our findings, where we show the proportion of GP surgeries prescribing broad-spectrum antibiotics is higher in more affluent areas.

Another study by Mölter and colleagues, who analysed antibiotic prescribing by GP practice in England, identified spatial clusters of high and low spots of prescribing (so-called ‘hot’ and ‘cold’ spots) [27]. The work showed that the distribution of antibiotic prescribing was heterogeneous, with the majority of the hot spots located in the North of England. Our results confirm this as we found that, when controlling for demographic, and health need variables, highest levels of prescribing were found in the North East, and East of England; lowest levels of antibiotic prescribing were consistently found in London. Overall, our study builds on previous research, and shows that even after using a proxy indicator to control for health need, there is evidence of significant inequalities for those in the bottom three deciles compared to those living in the least deprived decile.

Given the emphasis and strategic importance – at both a national and international level – of developing and implementing antibiotic stewardship polices, our findings are timely and potentially have important implications for policymakers. The national strategy of reducing the use of antibiotics appears to be working, given that our data shows a reduction in antibiotic items per STAR-PU each year. This is in line with other work that also shows a similar reduction in antibiotic prescribing [28]. However, current prescribing targets only account for age and sex of the population served. For example, men aged 75 years and above are weighted as 1.0, while men aged between 35 and 44 years are weighted as 0.3; in contrast, women aged 75 years and above are weighted as 1.3, while women aged between 35 and 44 years are weighted as 0.6 [29]. The weighting does not consider any measure of area-level deprivation or related-health need. Our results suggest that it would be prudent for any future antibiotic prescribing targets to acknowledge that GP surgeries located in the most deprived communities are likely to have a higher health need in terms of antibiotic use, and account for this in their targets. Local antimicrobial stewardship approaches should also be considered at an area level to account for specific pressures and needs. In addition, any future revision of the prescribing measure items per STAR-PU should also consider incorporating a measure of deprivation into their weighting. This finding was also echoed by Pouwels and colleagues [7], who suggest it would be advantageous to avoid the same prescribing targets for all GP practices, or it would be important to develop alternative approaches that encompass additional predictors of antibiotic prescribing. This is similar to the way in which NHS funding allocation policy incorporates deprivation [30].

### Strengths and limitations

We believe our modelling results are robust; the residuals are normally distributed, and the VIF ratio is low (2.07) therefore homoscedasticity and multicollineararity are not considered problematic. However, we do acknowledge there are a number of limitations to our work. Firstly, we only assessed the amount of antibiotic prescribing according to items per STAR-PU; we did not consider the appropriateness of prescribing, nor did we consider the patient characteristics for whom the antibiotics were prescribed. It is possible, therefore, that the higher antibiotic prescribing observed in the most deprived areas were prescribed either unnecessarily or inappropriately. Indeed, Smith and colleagues showed that in an English primary care setting, most antibiotics are prescribed for conditions that only sometimes required antibiotics, which was dependent on patient specific indicators (e.g. co-morbidity) [31]. Our study does not account for this. It would be prudent, therefore, for future work to assess the appropriateness of antibiotic prescribing for GPs located in deprived areas. Our data was obtained free of charge from the NHS Business Service Authority at the GP practice level; data at the patient – or individual prescriber level – was not available through this route. Collecting patient-level data, and making it freely available for research or system improvement purposes without expensive subscriptions would be advantageous as it would allow the assessment of prescribing appropriateness. A further limitation of our work is in relation to how we adjusted for health need: in our linear regression model, we only used COPD and diabetes prevalence as proxies for a health need measure. In addition to COPD and diabetes prevalence, there are other reasons that may contribute to increased susceptibility of developing a bacterial infection, including poor living conditions [32], reduced vaccination uptake [33], poor nutrition [34], and higher incidence of smoking [35]. Frailty may also be associated with increased antibiotic prescribing, which could have been accounted for using the eFrailty index [36]. These additional factors were not accounted for in our analysis. In addition, we also only analysed 4 years of data as prior to this, there were changes in the methodology of recording of prescribing data, making it challenging to investigate longer-term trends in antibiotic prescribing using our data sources. Furthermore, our study was ecological in design: thus, we acknowledge that relationships that apply at an area-level do not necessarily apply at an individual level – such an assumption would be committing the ecological fallacy. As such, there is no measure to link antibiotics prescribed at a GP level with patients who receive those antibiotics at an individual level.

## Conclusion

Despite a reduction over time in antibiotic prescribing, there is still significant variation by area-level deprivation and English region in prescribing levels. Our analysis demonstrated higher overall antibiotic prescribing in more deprived areas, although the proportion of GP surgeries prescribing broad spectrum antibiotics was higher in more affluent areas. Health need is an important consideration in antibiotic prescribing, but it did not explain all of the variation. Future antibiotic prescribing targets should account for local factors, including deprivation, to ensure GP practices located in the most deprived communities are not inappropriately penalised.

## Supplementary information

**Additional file 1: Supplementary Figure 1.** Histogram of antibiotic STAR-PU before (A) and after (B) trimming top and bottom 1% of data.

**Additional file 2: Supplementary Figure2.** GP practice concentration by IMD decile.

## Data Availability

The data analysed in this study were obtained from the NHS Business Services Authority ePACT2 system. Under our data use agreement, we are unable to share the data, although they may be obtained through request to the NHS Business Services Authority.

## Abbreviations

COPD: Chronic Obstructive Pulmonary Disease
CCG: Clinical Commissioning Group
GP: General Practitioner
IMD: Index of Multiple Deprivation
LSOA: Lower Layer Super Output Area
NHSBSA: NHS Business Services Authority
SIMD: Scottish Index of Multiple Deprivation
STAR-PU: Specific Therapeutic Group Age-sex weightings Related Prescribing Units
VIF: Variance of inflation
WHO: World Health Organisation
