# Supplementary figures and images for: An examination of trends in antibiotic prescribing in primary care and the association with area-level deprivation in England
Source: BMC Public Health. 2020 Aug 3;20:1148. doi: 10.1186/s12889-020-09227-x (PMC7397662; doi:10.1186/s12889-020-09227-x)

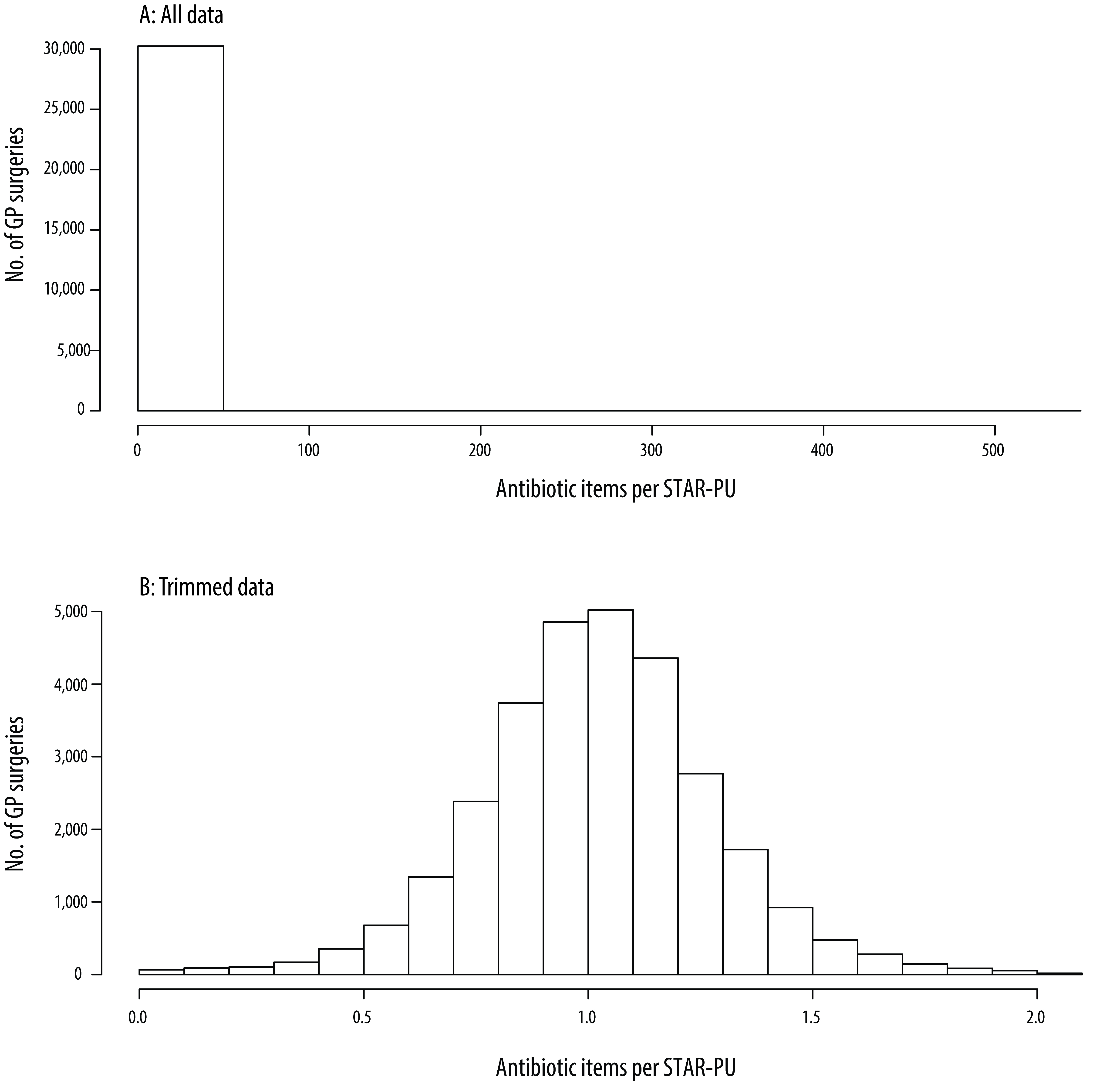

Supplement: Supplementary file 1 — Additional file 1: Supplementary Figure 1. Histogram of antibiotic STAR-PU before (A) and after (B) trimming top and bottom 1% of data. [file 12889_2020_9227_MOESM1_ESM.jpg]

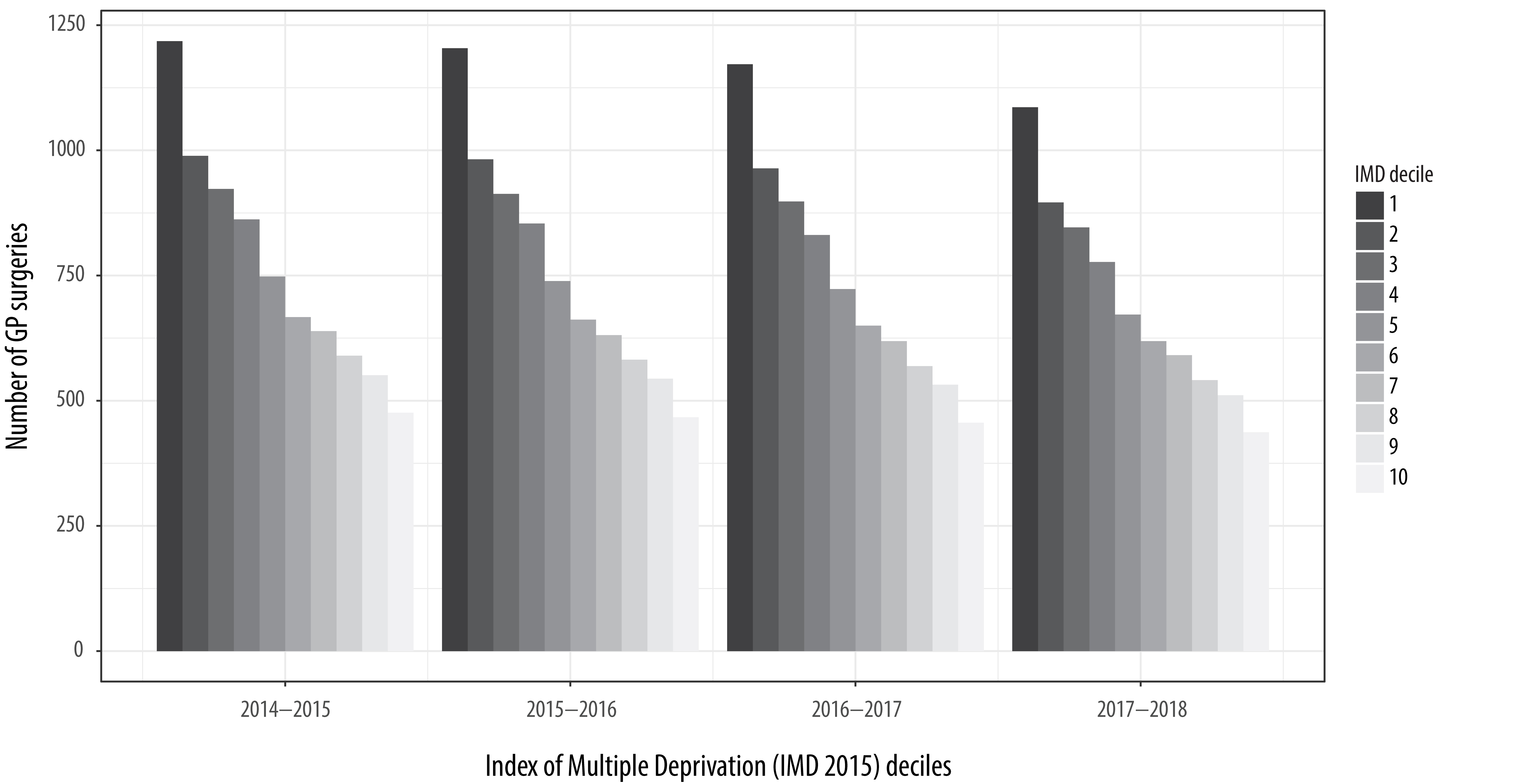

Supplement: Supplementary file 2 — Additional file 2: Supplementary Figure2. GP practice concentration by IMD decile. [file 12889_2020_9227_MOESM2_ESM.jpg]
